# Characterization of the complete mitochondrial genome of the cloacal tapeworm *Cloacotaenia megalops* (Cestoda: Hymenolepididae)

**DOI:** 10.1186/s13071-016-1782-0

**Published:** 2016-09-05

**Authors:** Aijiang Guo

**Affiliations:** 1 State Key Laboratory of Veterinary Etiological Biology, Key Laboratory of Veterinary Parasitology of Gansu Province, Lanzhou Veterinary Research Institute, Chinese Academy of Agricultural Sciences, Lanzhou, 730046 Gansu Province People’s Republic of China; 2 Jiangsu Co-innovation Center for Prevention and Control of Important Animal Infectious Diseases and Zoonoses, Yangzhou, 225009 Jiangsu Province People’s Republic of China

**Keywords:** Tapeworm, Cestoda, Hymenolepididae, *Cloacotaenia megalops*, Mitochondrial genome, Phylogenetic analyses

## Abstract

**Background:**

The cloacal tapeworm *Cloacotaenia megalops* (Hymenolepididae) is one of the most common cestode parasites of domestic and wild ducks worldwide. However, limited information is available regarding its epidemiology, biology, genetics and systematics. This study provides characterisation of the complete mitochondrial (mt) genome of *C. megalops*.

**Methods:**

The complete mt genome of *C. megalops* was obtained by long PCR, sequenced and annotated.

**Results:**

The length of the entire mt genome of *C. megalops* is 13,887 bp; it contains 12 protein-coding, 2 ribosomal RNA and 22 transfer RNA genes, but lacks an *atp*8 gene. The mt gene arrangement of *C. megalops* is identical to that observed in *Anoplocephala magna* and *A. perfoliata* (Anoplocephalidae), *Dipylidium caninum* (Dipylidiidae) and *Hymenolepis diminuta* (Hymenolepididae), but differs from that reported in taeniids owing to the position shift between the tRNA (L1) and tRNA (S2) genes. The phylogenetic position of *C. megalops* was inferred using Maximum likelihood and Bayesian inference methods based on the concatenated amino acid data for 12 protein-coding genes. Phylogenetic trees showed that *C. megalops* is sister to *Anoplocephala* spp. (Anoplocephalidae) + *Pseudanoplocephala crawfordi* + *Hymenolepis* spp. (Hymenolepididae) indicating that the family Hymenolepididae is paraphyletic.

**Conclusions:**

The complete mt genome of *C. megalops* is sequenced. Phylogenetic analyses provided an insight into the phylogenetic relationships among the families Anoplocephalidae, Hymenolepididae, Dipylidiidae and Taeniidae. This novel genomic information also provides the opportunity to develop useful genetic markers for studying the molecular epidemiology, biology, genetics and systematics of *C. megalops*.

**Electronic supplementary material:**

The online version of this article (doi:10.1186/s13071-016-1782-0) contains supplementary material, which is available to authorized users.

## Background

The cloacal tapeworm, *Cloacotaenia megalops* Nitzsch in Creplin, 1829 (Cestoda: Hymenolepididae), is one of the most common hymenolepidid tapeworms parasitising waterfowl, with a global distribution. The life-cycle of this tapeworm is complex. Seed shrimpos (Ostracoda) act as intermediate hosts and many waterfowl species (including ducks, geese and swans) serve as definitive hosts [1]. In China, *C. megalops* is considered as a predominant cestode species in ducks and geese [2, 3].

Comparison of entire mitochondrial (mt) genomes has been used for reconstructing phylogenetic relationships among parasitic Platyhelminthes [4, 5], including cestodes [6–11]. Cestode mt genomes usually encode 36 genes, including 12 protein-coding genes, 2 ribosomal RNA (rRNA) genes and 22 transfer RNA (tRNA) genes [6–11]. Cestoda is a large class of parasitic flatworms with many species representing a health danger for animals and humans worldwide. Despite the availability of advanced DNA technologies and bioinformatic methods, there is still a paucity of knowledge of mt genomes for many tapeworms of socioeconomic importance, such as the members of the family Hymenolepididae. Although complete mt genomes are available for *Hymenolepis diminuta* [8], *H. nana* (=*Rodentolepis nana* or *Vampirolepis nana*) [9], and *Pseudanoplocephala crawfordi* [10], no mt genomes are available from the genus *Cloacotaenia*. Furthermore, little is known about the epidemiology, genetics and biology of the type-and only species of this genus, *C. megalops*.

The taxonomic status of *C. megalops* has been controversial for many years, and is still debated. Czaplinski & Vaucher [12] considered *Cloacotaenia* a synonym of *Hymenolepis* but Makarikov et al. [13] have recently restored the independent status of the genus *Cloacotaenia* based on remarkable morphological differences between *C. megalops* and *Hymenolepis* (*sensu stricto*). To tackle these issues, in the present study, the complete mt genome of *C. megalops* was determined and its phylogenetic relationships with selected cestode species were inferred based on analysis of the concatenated mt amino acid sequences.

## Methods

### Parasites and DNA extraction

*Cloacotaenia megalops* were collected from the cloaca of ducks from a small abattoir in Xinjiang Uygur Autonomous Region, China. The adult tapeworm was isolated from cloaca of a duck. Cestode identification was conducted by morphological criteria including the features of the scolex and mature and gravid proglottids [14]; the scolex was observed in stereoscan photographs and mature and gravid proglottids were examined after hematoxylin staining. The remaining fragment was fixed in 70 % alcohol and stored at -20 °C until use. Total genomic DNA was extracted from one of these specimens using Tissue DNA Kit (OMEGA, Doraville, USA) according to the manufacturer’s instructions.

### PCR amplification and sequencing

Three pairs of PCR primers (Additional file 1: Table S1) were designed based on well-conserved regions within the mt genomes of tapeworms [6, 7]. These primers were used to amplify three overlapping segments of the complete mt genome of *C. megalops* by long PCR technology. Long PCR reactions (50 μl) were conducted in 5.0 μl 10× LA Mixture (Takara), 10 pmol of each primer (1 μl), 1.5 μl of DNA sample and 41.5 μl of H_2_O in a thermocycler (Eppendorf, Hamburg, Germany) under the following conditions: 94 °C for 5 min (initial denaturation), followed by 35 cycles of 98 °C for 10 s (denaturation), 50 °C for 20 s (annealing), and 68 °C for 8 min (extension), and with a final extension step at 68 °C for 10 min. Amplicons were examined on 0.8 % agarose gels stained with ethidium bromide. PCR products were subsequently sent to Sangon Biotech Co. Ltd. (Shanghai, China) for sequencing using a primer-walking strategy.

### Sequence analyses

Sequences were assembled using CAP3 Server online. The complete mt genome of *C. megalops* was aligned against the complete mt genome sequences of *H. diminuta* and *A. perfoliata* using the computer program MAFFT 7.122 [15] to identify gene boundaries. Each gene was translated into its amino acid sequence using the flatworm mt genetic code (Translation table 9) in MEGA 5 [16]. The translation start and stop codons were identified based on the similarity of the gene lengths and usual codons between *H. diminuta* and *A. perfoliata* mt genomes. Twenty-two tRNA genes were predicted using the program tRNAscan-SE [17] and then confirmed by recognizing anticodon sequences and potential secondary structures by visual inspection, and two rRNA genes were identified by comparison with that of *H. diminuta* and *A. perfoliata* [7, 8].

### Phylogenetic analyses

A total of 20 tapeworm species were selected for phylogenetic analyses using one trematode *Schistosoma japonicum* (GenBank accession number NC_002544) as the outgroup [18]. The 12 amino acid sequences of protein-coding genes were aligned independently using MAFFT 7.122. Ambiguously aligned sites and regions were excluded using Gblocks (http://molevol.cmima.csic.es/castresana/Gblocks_server) [19]. Phylogenetic analyses were performed using Bayesian inference (BI) and Maximum likelihood (ML) methods. The Akaike information criterion as implemented in ProtTest 2.4 [20] was used to choose the most suitable model of evolution. BI was performed in MrBayes using the MtArt + I + G + F model of evolution. BI was set up to perform two runs, each of four simultaneous chains for the Monte Carlo Markov Chain. In each run, the number of generations was set to 1,000,000 and a tree was sampled every 100 generations in MrBayes 3.1.1 [21]; the average standard deviation of split frequencies of less than 0.01 and the potential scale reduction factor approaching 1 were used to ensure the convergence of the two runs. The first 25 % of the trees were discarded as ‘burn-in’. A 50 % majority rule consensus tree was used to calculate Bayesian posterior probabilities (Bpp). ML analysis was conducted using PhyML 3.0 [22]. A BioNJ tree was used as a starting tree to search for the ML tree with the MtArt + I + G model of evolution. The subtree pruning and regrafting method was chosen. The middle of each discretized substitution rate class was determined using the median. ML analyses were checked on the basis of 100 bootstrap replicates (Bf). Phylograms were drawn using the program FigTree v.1.4 (http://tree.bio.ed.ac.uk/software/figtree).

## Results and discussion

### Features of the *C. megalops* mt genome

**Fig. 1:**
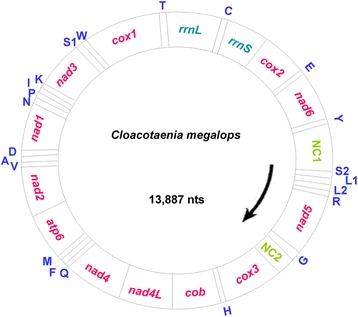
The organization of mitochondrial genome of *Cloacotaenia megalops*. All genes are transcribed in the same direction and the 22 tRNA genes are shown by a single-letter abbreviation of their corresponding amino acid. The two leucine tRNA genes are designated by L_1_ (CUN) and L_2_ (UUR), respectively, and two serine tRNA genes by S_1_ (AGN) and S_2_ (UCN), respectively. Gene scaling is only approximate

The complete mt genome of *C. megalops* is a 13,887 bp (KU641017) long circular DNA molecule (Fig. 1). All 36 genes expected for tapeworm mt genomes have been identified. This AT rich (71.6 %) mt genome includes 12 protein-coding genes (*atp*6, *cox*1–3, *cyt*b, *nad*1–6 and *nad*4L), 22 tRNA genes and two rRNA genes, but lacks an *atp*8 gene (Table 1). Thirty-six mt genes are transcribed from the same direction (Fig. 1). The size of *C. megalops* mt genome is similar to other tapeworm mt genomes, such as *H. diminuta* (13,900 bp) [8], *H. nana* (13,764 bp) [9] and *P. crawfordi* (14,192 bp) [10]. The mt gene arrangement of *C. megalops* is identical to that in *Anoplocephala magna* and *A. perfoliata* (Anoplocephalidae), *Dipylidium caninum* (Dipylidiidae) and *Hymenolepis diminuta* (Hymenolepididae), but differs from that in taeniids owing to the position shift between the tRNA (L1) and tRNA (S2) genes. The nucleotide composition of the entire mt genome of *C. megalops* is A = 26.4 %, T = 45.2 %, G = 18.7 % and C = 9.7 %.

**Table 1:** Organization of *Cloacotaenia megalops* mitochondrial genome

Gene/region	Position	Size (bp)	Start codon	Stop codon
*cox*1	1–1593	1593	ATG	TAA
*tRNA*-*Thr* (T)	1574–1637	64	–	–
*rrn*L	1638–2596	959	–	–
*tRNA*-*Cys* (C)	2597–2660	64	–	–
*rrn*S	2661–3382	722	–	–
*cox*2	3383–3961	579	ATG	TAG
*tRNA*-*Glu* (E)	3963–4029	67	–	–
*nad*6	4033–4482	450	ATG	TAA
*tRNA*-*Tyr* (Y)	4487–4548	62	–	–
Non-coding region (NC1)	4549–4759	211	–	–
*tRNA*-*Ser*UCN (S2)	4760–4824	65	–	–
*tRNA*-*Leu*CUN (L1)	4841–4904	64	–	–
*tRNA*-*Leu*UUR (L2)	4907–4971	65	–	–
*tRNA*-*Ar*g (R)	4972–5027	56	–	–
*nad*5	5031–6602	1572	ATG	TAA
*tRNA*-*Gly* (G)	6609–6680	72	–	–
Non-coding region (NC2)	6681–7126	446	–	–
*cox*3	7127–7780	654	ATG	TAG
*tRNA*-*His* (H)	7771–7837	67	–	–
*cytb*	7841–8932	1092	ATG	TAG
*nad*4L	8936–9196	261	ATG	TAA
*nad*4	9157–10,410	1254	ATG	TAG
*tRNA*-*Gln* (Q)	10,411–10,473	63	–	–
*tRNA*-*Phe* (F)	10,475–10,533	59	–	–
*tRNA*-*Met* (M)	10,532–10,596	65	–	–
*atp*6	10,600–11,115	513	ATG	TAG
*nad*2	11,121–11,999	879	ATG	TAG
*tRNA*-*Val* (V)	12,009–12,072	64	–	–
*tRNA*-*Ala* (A)	12,073–12,135	63	–	–
*tRNA*-*Asp* (D)	12,149–12,211	63	–	–
*nad*1	12,215–13,105	891	ATG	TAG
*tRNA*-*Asn* (N)	13,105–13,169	65	–	–
*tRNA*-*Pro* (P)	13,184–13,247	64	–	–
*tRNA*-*Ile* (I)	13,248–13,308	61	–	–
*tRNA*-*Lys* (K)	13,320–13,384	65	–	–
*nad*3	13,388–13,741	354	ATG	TAG
*tRNA*-*Ser*AGN (S1)	13,749–13,809	61	–	–
*tRNA*-*Trp* (W)	13,820–13,884	65	–	–

### Annotation

A total of 3352 amino acids are encoded in the *C. megalops* mt genome. The aggregate length of all of the 12 protein-coding genes is 10,092 bp. In terms of the length of individual protein-coding genes, *cox*1 gene is the largest (1593 bp) and *nad*4L gene is the shortest (261 bp). In this mt genome, all protein-coding genes use ATG as start codon (Table 1). All protein-coding genes have complete termination codons (TAA and TAG) (Table 1). However, some studies have indicated that the incomplete termination codons T or TA are present in the protein-coding genes of some tapeworm mt genomes [7, 23]. A total of 22 tRNA (ranging from 56 to 72 nucleotides in length) genes were identified. Their predicted secondary structures (not shown) are similar to those in *H. diminuta* and *A. perfoliata* [8]. The tRNA-Cys gene separates *rrn*L from *rrn*S. The size of the *rrn*L gene is 959 bp and the size of the *rrn*S gene is 722 bp (Table 1). One larger non-coding region (NC2; 446 bp) is located between the tRNA-Gly and *cox*3 genes, and one shorter non-coding region (NC1; 221 bp) is located between the tRNA-Tyr and tRNA-Ser genes (Table 1; Fig. 1). In the NC1 region, there were two sets of short inverted repeats and one set of long inverted repeats (33 bp), each of them could be folded into a stem-loop hairpin structure (Additional file 2: Figure S1A). The NC2 region consists of six identical tandem repeats with 31 bp sequences (Additional file 2: Figure S1B). Similar stable hairpin structures and tandem repeats in *C. megalops* may play the same role as those in vertebrates, which have been shown to initiate replication and transcription [24].

### Sequence comparisons

Pairwise comparisons of *C. megalops* mt protein-coding genes with those of three other hymenolepidid tapeworms revealed 12.9–35.3 % differences in the nucleotide sequences, and 10.6–43.6 % differences in amino acid sequences (Table 2). Among twelve protein-coding genes, *cox*1 and *cytb* genes were relatively conserved whilst *nad*5 and *nad*6 genes were the most different in all four species (Table 2). These results are useful to design primers to capture high sequence variability within and between mt genes of these species as genetic markers for population genetics and diagnostics.

**Table 2:** Nucleotide and/or deduced amino acid (aa) sequence differences of the protein-coding and two ribosomal RNA genes of the mt genomes of *Cloacotaenia megalops* (CM), *Hymenolepis nana* (HN), *Hymenolepis diminuta* (HD) and *Pseudanoplocephala crawfordi* (PC)

Gene/region	Nucleotide length (bp)				Nucleotide difference (%)						Number of aa				aa difference (%)					
	CM	HN	HD	PC	CM *vs* HD	CM *vs* HN	CM *vs* PC	HN *vs* HD	HN *vs* PC	HD *vs* PC	CM	HN	HD	PC	CM *vs* HN	CM *vs* HD	CM *vs* PC	HN *vs* HD	HN *vs* PC	HD *vs* PC
*atp*6	513	516	516	516	30.0	30.2	30.4	26.9	28.2	21.9	170	171	171	171	27.5	29.2	30.4	30.4	32.7	33.1
*nad*1	891	894	891	891	25.6	25.7	25.8	21.7	19.8	16.4	296	297	296	296	23.6	25.7	25.0	18.9	18.2	12.2
*nad*2	879	885	882	897	31.7	32.1	32.3	27.0	27.1	24.7	292	294	293	298	37.7	39.4	41.4	34.5	35.6	24.7
*nad*3	354	348	348	348	31.3	29.0	28.2	25.3	27.3	21.8	117	115	115	115	36.5	38.3	37.4	22.6	29.6	19.1
*nad*4	1254	1209	1230	1230	31.1	33.4	32.4	29.6	33.9	25.5	417	402	409	409	33.3	36.9	35.9	32.7	34.2	25.4
*nad*4L	261	261	261	261	27.6	26.4	27.6	20.7	20.3	16.1	86	86	86	86	30.2	32.6	29.1	20.9	21.9	19.8
*nad*5	1572	1575	1575	1575	31.7	35.3	34.2	31.2	31.7	25.9	523	524	524	524	33.3	38.6	34.1	35.4	36.6	26.1
*nad*6	450	459	459	459	34.0	33.6	32.7	32.2	30.3	24.6	149	152	152	152	42.3	43.2	43.6	39.1	39.7	24.3
*cox*1	1593	1584	1552	1582	23.2	23.4	22.4	20.6	19.4	17.3	530	527	517	527	15.9	18.6	16.7	16.3	16.2	10.6
*cox*2	579	573	579	579	26.3	27.7	26.9	26.4	28.3	18.3	192	190	192	192	21.9	26.8	24.5	26.3	28.4	13.0
*cox*3	654	645	651	651	32.0	31.7	30.7	29.8	30.1	20.5	217	214	216	216	41.6	42.5	38.3	34.6	31.3	26.4
*cytb*	1092	1098	1098	1095	25.3	23.8	25.4	23.0	23.4	20.3	363	365	365	364	22.9	20.9	23.8	17.3	20.9	15.7
*rrn*S	722	710	709	724	18.7	19.8	20.5	16.5	17.0	12.9	–	–	–	–	–	–	–	–	–	–
*rrn*L	959	967	967	963	23.3	20.7	24.0	21.8	23.2	19.2	–	–	–	–	–	–	–	–	–	–

### Phylogenetic analyses

**Fig. 2:**
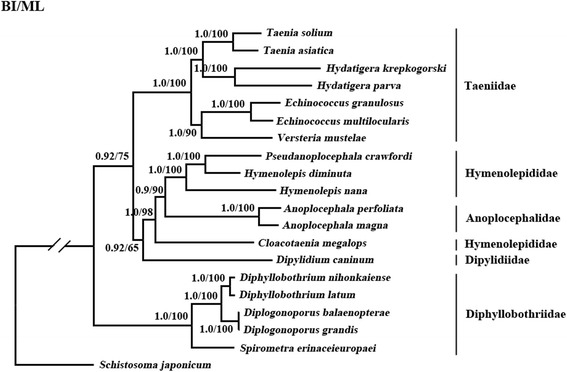
Phylogenetic relationships among 19 species of tapeworms. Phylogenetic tree was inferred by Bayesian inference and Maximum likelihood analysis from deduced amino acids of 12 protein-coding genes using *Schistosoma japonicum* as the outgroup. Bayesian posterior probability (Bpp) and bootstrapping frequency (Bf) values are shown at nodes

Phylogenetic analyses showed three distinct groups with high statistical support (Bpp = 1.0; Bf = 98 %) with *C. megalops* as a sister taxon to *Anoplocephala* spp. (Anoplocephalidae) + *P. crawfordi* + *Hymenolepis* spp. (Hymenolepididae) (Fig. 2), indicating that the family Hymenolepididae is paraphyletic. The result is consistent with the maximum likelihood analysis in the study by von Nickisch-Rosenegk et al. [25] in which 12S rDNA data of a wider set of taxa representing a larger number of families. The families Taeniidae, Anoplocephalidae and Diphyllobothriidae were monophyletic with maximum support in all analyses (Bpp = 1.0; Bf = 100 %) (Fig. 2), in agreement with previous studies [7, 9, 26]. In addition, our results show that *H. diminuta* is more closely related to *P. crawfordi* than to *H. nana* (Fig. 2).

In this study, the phylogenetic tree revealed that *C. megalops* is distantly related to the genus *Hymenolepis*, supporting the view of Makarikov et al. [13] who preferred to retain the independent status of *Cloacotaenia* based on the morphological characteristics. The present results also suggest that *Pseudanoplocephala crawfordi* is a member of the genus *Hymenolepis* as shown in previous studies [10, 27]. *Hymenolepis nana* is one of the most common tapeworms infecting humans and rodents. However, there is confusion regarding the nomenclature of this species [28]. A taxonomic revision of hymenolepidids with armed rostellae was suggested by Vaucher [29] in which *Hymenolepis nana* was regarded as a member of the genus *Rodentolepis*. This classification is currently accepted by some cestode taxonomists [12]. Despite the nomenclature being revised, the name *H. nana* persists in textbooks and medical papers [30]. Additionally, whether the Anoplocephalidae should be nested among species of the Hymenolepididae should also be rigorously evaluated in further studies based on more extensive taxon sampling of hymenolepidids. This will also help a better understanding of the evolution of hymenolepidid cestodes and a re-evaluation of the morphological traits employed in their systematics.

## Conclusions

The complete mt genome of *C. megalops* is characterised. Phylogenetic analyses of the concatenated amino acid sequence dataset for 12 protein-coding mt genes of *C. megalops* and selected cestode representatives indicated that the family Hymenolepididae is paraphyletic. This mt genome provides a unique genetic marker for studying the molecular biology, genetics and systematics of *C. megalops*.

## Additional files

Additional file 1:
**Table S1.** Primers used to amplify PCR fragments for *Cloacotaenia megalops*. (DOC 37 kb) Additional file 2:
**Figure S1.** Putative secondary structures for the two non-coding regions in *Cloacotaenia megalops* mtDNA. The NC1 (A) consists of two identical repeats of 34 nt shown in the box. The NC2 region (B) consists of six identical tandem repeats of a 31 nt sequence and part of the seventh repeat (10 nt). Arrows represent inverted repeats. (DOC 330 kb)

## Abbreviations

BI: Bayesian inference
Bpp: Bayesian posterior probabilities
ML: Maximum likelihood
Mt: Mitochondrial
