# Supplementary material for: Characterization of the complete mitochondrial genome of the cloacal tapeworm Cloacotaenia megalops (Cestoda: Hymenolepididae)
Source: Parasit Vectors. 2016 Sep 5;9(1):490. doi: 10.1186/s13071-016-1782-0 (PMC5011890; doi:10.1186/s13071-016-1782-0)
Supplement: Additional file 1: — Table S1. Primers used to amplify PCR fragments for Cloacotaenia megalops. (DOC 37 kb) [file 13071_2016_1782_MOESM1_ESM.doc]

**Additional file 1**

**Characterization of the complete mitochondrial genome of** **the cloacal tapeworm *Cloacotaenia megalops* (Cestoda: Hymenolepididae)**

Aijiang Guo

**Additional file 1: Table S1.** Sequences of primers used to amplify PCR fragments from *Cloacotaenia megalops*

| Primer | Sequence (5’ to 3’) |
| --- | --- |
| *nad*1-*rrn*S F | CAGTTTCGTAAGGGTCCTAATAAG |
| *nad*1-*rrn*S R | AAAGTTACCTTGTTACGACTTACCTC |
| *rrn*S-*nad*5 F | AGGGGATAGGACACAGTGCCAGCAT |
| *rrn*S-*nad*5 R | GGAAATCTGGCGCTCTTAGT |
| *nad*5-*nad*1 F | TGTATGAGTTAGTTTTAAGCATTAATTA |
| *nad*5-*nad*1 R | CCCTTCTTGAAGTTAACAGCAT |
