# Supplementary material for: Characterization of the complete mitochondrial genome of the cloacal tapeworm Cloacotaenia megalops (Cestoda: Hymenolepididae)
Source: Parasit Vectors. 2016 Sep 5;9(1):490. doi: 10.1186/s13071-016-1782-0 (PMC5011890; doi:10.1186/s13071-016-1782-0)
Supplement: Additional file 2: — Figure S1. Putative secondary structures for the two non-coding regions in Cloacotaenia megalops mtDNA. The NC1 (A) consists of two identical repeats of 34 nt shown in the box. The NC2 region (B) consists of six identical tandem repeats of a 31 nt sequence and part of the seventh repeat (10 nt). Arrows represent inverted repeats. (DOC 330 kb) [file 13071_2016_1782_MOESM2_ESM.doc]

**Additional file 2**

**Characterization of the complete mitochondrial genome of** **the cloacal tapeworm *Cloacotaenia megalops* (Cestoda: Hymenolepididae)**

Aijiang Guo

**
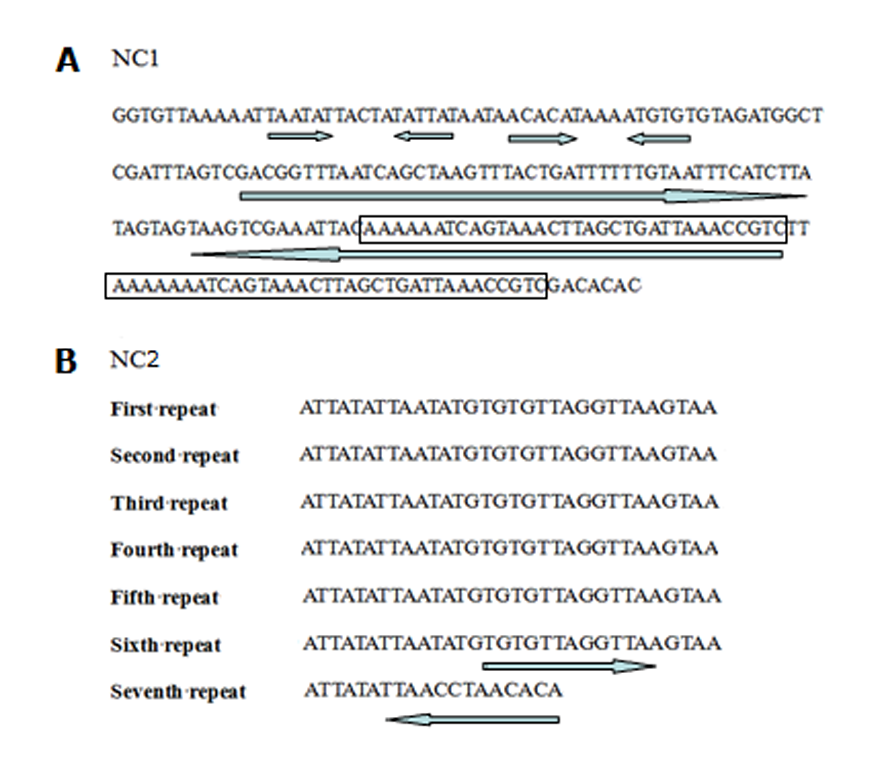
**

**Additional file 2: Figure S1.** Putative secondary structures for the two non-coding regions in *Cloacotaenia megalops* mtDNA. The NC1 (A) consists of two identical repeats of 34 nt shown in the box. The NC2 region (B) consists of six identical tandem repeats of a 31-nt sequence and part of the seventh repeat (10 nt). Arrows represent inverted repeats
